# Implementation of a Multifactorial Strategy Including Direct Bedding Is Associated With a Rapid and Sustained Reduction in Left Without Being Seen

**DOI:** 10.7759/cureus.16209

**Published:** 2021-07-06

**Authors:** Jennifer Martin, Thomas Brunnell, Matthew Neulander, Emily Ryan, Elizabeth Schiller, Megan Smith, Steven Wolf, Patti LaMonica, Kelly Chevalier, Brenda Theriaque, Reginald Eadie

**Affiliations:** 1 Emergency Medicine, St. Francis Hospital and Medical Center, Hartford, USA

**Keywords:** efficiency, ed throughput, direct bedding, ed operations, lwbs, quality, left without being seen

## Abstract

Objective

Improve left without being seen (LWBS) in our high volume, tertiary care trauma center. Prior to intervention, our LWBS rate was 4.4%. Including a direct bedding strategy, we successfully reduced our LWBS to <1%.

Design and method

We utilized a retrospective before and after model. We hired a clinical documentation specialist and tracked several metrics. These included daily census, admission rates, and door to provider, door to room, average boarding, and door to disposition times. Data were collected and disseminated daily. Reports were shared at organization quality meetings. Simultaneously, we implemented the direct bedding initiative in conjunction with quick registration. To accommodate higher numbers of patients and expediate movement to care spaces, all patient spaces were clearly designated and labeled.

Results

Direct bedding began in September 2015 and our LWBS was 4.4%. One-year post-intervention, our LWBS was <2%. Within four years, it was <0.5%. The LWBS rate for each year, 2016 to 2019, was significantly lower than the control period (p < 0.01) (2015 up to September). Improvement was also seen in door-to-provider time and with patient experience scores.

Conclusion

Our multifactorial approach was associated with a profound and sustained reduction in LWBS over a short time period.

## Introduction

Left without being seen (LWBS) has been identified as an important quality metric in many emergency departments (ED). The factors which influence LWBS are multiple and complex. The patient population who leave the ED prior to evaluation are at significant risk for poor outcomes [1]. The risks include but are not limited to increased morbidity and mortality as well as an increased rate of hospital readmission, which represent similar risks to that of patients who leave against medical advice [1]. This increased risk, not surprisingly, has become a focus for the Joint Commission [2-5]. Other national agencies, including the Institute of Medicine, the Agency for Healthcare Research and Quality Improvement, and the Institute for Healthcare Improvement have all recognized that ED efficiency, particularly LWBS, significantly impacts patient outcomes [2-5]. As of January 2012, the annual reporting of LWBS rates as a quality metric is required by the Centers for Medicare and Medicaid Services [6]. LWBS may also represent a significant negative economic impact on hospitals in the form of lost revenue and exposure to increased risk [7]. To date, these authors have not yet found decisive data to demonstrate the extent of this cost or exposure. However, strategies that reduce LWBS would have multiple benefits for patients, emergency departments, and hospital organizations.

LWBS has often been identified as a problem related to suboptimal ED efficiency. Approaches to decrease LWBS include placing a provider in triage, implementing nursing order protocols, streamlined communication between the ED/inpatient units, leasing mobile units to act as fast-track departments, and variations of direct bedding [6-15]. The provider in triage approach not only decreases LWBS but also increases ED throughput metrics especially in large urban hospitals [12]. Direct bedding refers to a strategy in which the patient is rapidly placed into a bed or care area minimizing time spent in triage or the waiting area. This can result in significantly decreased wait times. Other areas of focus have included streamlining the patient registration process, decreasing lab and radiology turnaround times, and improving housekeeping processes [6,9]. Back-end processes have also been the focus of improvement. These include organizational efforts to decrease ED boarding as well as improving the inpatient discharge and follow-up processes. Some efforts to decrease LWBS may also lead to unintended consequences including leaving without complete treatment [10,12-16]. The complexity of the ED-to-discharge process, whether from an inpatient unit or directly discharged from the emergency department, has not been amenable to a single change improvement. In other words, as of this writing, there are few, if any, papers advocating a “single bullet” methodology to accomplish a decrease in LWBS [15,17].

Our hospital is an urban, tertiary-care, level 1 trauma center and teaching hospital located in Hartford, CT. We see approximately 95,000 annual ED patient visits. The ED treats a diverse population with high acuity. The admission rate is greater than 25%, with a large percentage admitted to monitored or critical care areas. In 2015, facing a crisis of increasing patient volumes, higher ED wait times, decreasing patient satisfaction scores, and an increase in LWBS rates, ED leadership sought a novel, expeditious, and implementable solution to decrease LWBS. Identifying LWBS as a critical metric related to patient and organizational well-being was key to process change. In an effort directed at reducing LWBS, our ED implemented several interventions which included a direct bedding strategy. Known colloquially as “pull-to-full,” we utilized this direct bedding strategy successfully to reduce our LWBS rate over a two-year period [18].

## Materials and methods

Our group utilized a retrospective, before and after observational model. We tracked and disseminated a large volume of operational data on a daily basis. As part of the improvement process, in September of 2015, our ED hired a clinical documentation specialist (CDS). In our institution, a CDS is a clinical provider whose role in the organization is to collect, analyze, and disseminate ED metrics gleaned from chart review and patient experience. In this case, the CDS is a nurse. With years of clinical experience as a bedside nurse in the emergency department, she transitioned to clinical documentation approximately ten years prior to joining our institution. During her time as a documentation specialist, she worked in the office setting and in a small community emergency department. In conjunction with our electronic medical record-keeping, we began circulating and posting daily metrics. These included door-to-provider time, door-to-room time, average boarding time, and LWBS rates. Press Ganey surveys were also collected and analyzed. Over a two-year course, beginning in September 2015 (the hiring of our CDS) through September 2017, the ED treated approximately 180,000 patients.

We employed a “pull-to-full” intervention. "Pull-to-full" describes a direct bedding process where patients are assigned directly to an exam room to allow for triage and subsequent evaluation by a provider thereby bypassing the traditional waiting room. Since this was a quality improvement project, we did not require institutional review board (IRB) approval or patient consent. Census data as well as the metrics collected on a daily basis were utilized to determine improvements in our LWBS rates. All ED personnel including physicians, physician assistants, nurses, medical assistants and technicians, registration personnel, as well as our volunteer staff were given some level of education regarding the implementation of the “pull-to-full” initiative. The amount and detail of the education was commensurate to staff position and involvement in the process. Education included daily safety huddles consisting of both education and group feedback, with rapid improvement events and process changes, as well as individual feedback. There were also monthly departmental meetings and physician meetings and all meeting minutes were distributed to all levels of staff. We collected and disseminated daily data. We conducted weekly, monthly, and quarterly quality meetings where this data was reviewed. Over the initial two-year investigational period, additional metrics including elopement rate, daily admission percentages, and ED boarding hours, were added to our internal quality reviews. Data reviews allowed staff to focus on the forward movement of patients from the reception/waiting areas to any usable patient care space or bed. At departmental meetings and safety huddles, the "pull-to-full" process was reinforced. Patients were to be moved from the entrance areas to a usable patient care space as quickly as possible.

“Quick Reg” (short for quick registration), a rapid method of inputting patient data into our electronic medical record system, was implemented. This occurred at the patient’s point of entry into the emergency department. Full registration was then completed at the patient’s assigned room, bed, or chair. Patient care spaces were maximized, utilizing hallway spaces and chairs in an effort to direct bed as many patients as possible. Although the ED has 67 physical rooms, in an effort to increase capacity, we utilized additional spaces including hallway locations, and chairs. These efforts raised our capacity to over 115 patient care spaces (with some flexibility for higher numbers in cases of disaster and surge).

While the "pull-to-full" intervention was the primary focus during the initial two-year period from December 2015 to December 2017, the organization was heavily focused on multiple metrics as listed above. In particular, door-to-provider time was emphasized in 2018 and 2019 with individual provider performance being tracked.

Two-tailed t-test analysis was utilized to determine statistical significance for the control period (January 2015 to September 2015) with the following intervention period.

## Results

**Figure 1:**
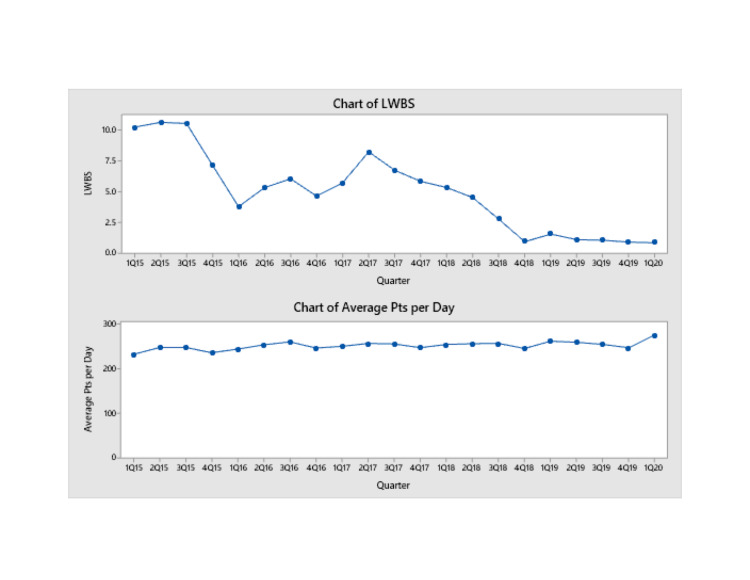
Charts of LWBS and patients seen per day. LWBS: left without being seen.

The ED LWBS direct bedding strategy (“pull to full”) began in September of 2015, when our LWBS rate was 4.4%. The direct bedding initiative was associated with an immediate and sustained decline in LWBS rates. ED volume remained stable, yet we were able to drop LWBS rates as low as 1.9% (Figure 1, bottom). There was a significant reduction in LWBS in 2016 compared to the control period of 2015 (p < 0.01). In addition, the LWBS rate for each subsequent year up to 2019 was also found to be statistically lower than the control period (p < 0.01).

**Figure 2:**
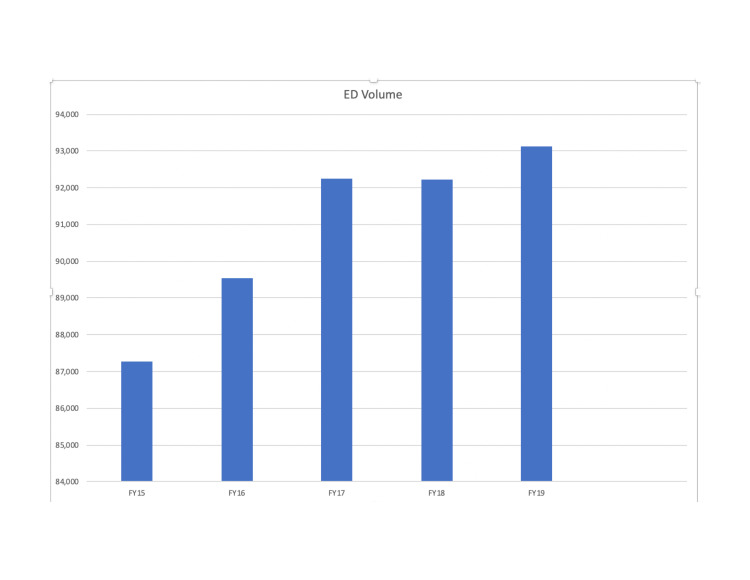
ED volume by fiscal year. ED: emergency department.

**Figure 3:**
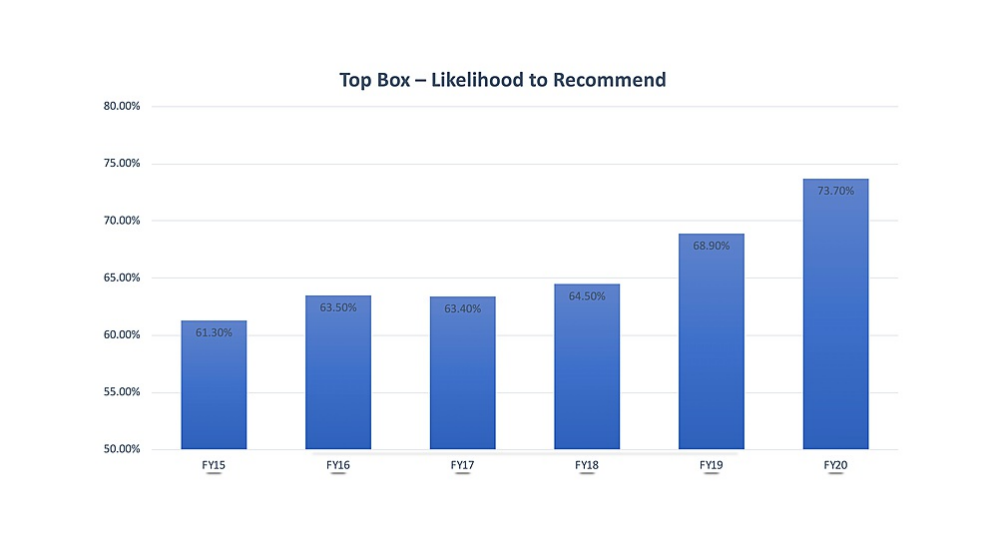
Likelihood to recommend.

**Figure 4:**
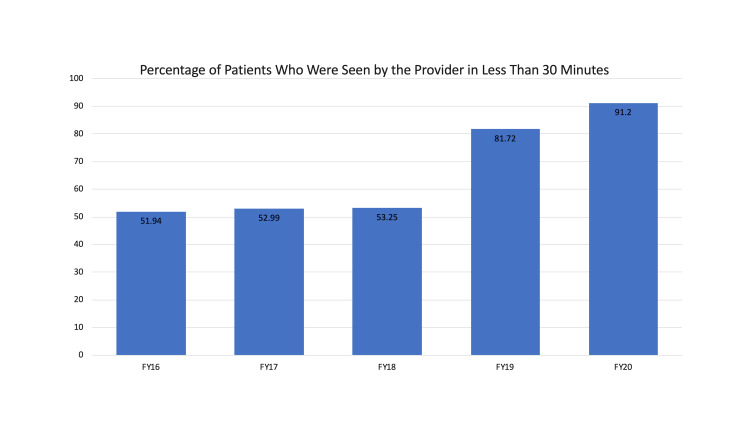
Percentage of patients who were seen by the provider in less than 30 minutes.

Of note, during our investigational period, there was a closure of two inpatient hospital units. The closure of the inpatient units resulted in a loss of up to 52 inpatient beds at a given time period. We believe that this resulted in an uptrend in LWBS from its nadir of 1.9% to the peak seen in the second quarter of 2017 (Figure 1, top). The closed units added a large number of boarded medical patients to the ED and limited our ability to effectively and efficiently directly bed patients. Given that our volume remained unchanged and we were boarding patients in the ED, we had less ability to bring patients directly back to a patient care area. Nonetheless, we continued to maintain improvements in our LWBS rates when compared to rates prior to implementation of "pull-to-full" (Figure 1). During this time, ED volumes were essentially unchanged (Figure 2). During the study period, we also observed an improvement in our overall rating of ED care as reflected in a rise in our Press Ganey scores (Figure 3). We additionally implemented efforts to see patients in a timely fashion, resulting in a greater proportion of patients being seen in less than 30 minutes (Figure 4). This was not our main outcome so we did not run statistical analysis.

## Discussion

LWBS is a significant healthcare quality metric that directly impacts the care provided to patients [16]. Increasing LWBS correlates with decreased patient safety and quality of care [17]. It represents a loss of income for the ED and the health care organization. Our ED implemented multiple interventions starting with a direct bedding strategy primarily to quickly and effectively decrease our LWBS. The results were rapid and dramatic. There were a large number of repercussions, some positive and some negative, involved with rapidly moving forward with "pull-to-full". The frequent collection and dissemination of ED metrics assisted in changing culture across the institution. It facilitated other processes to increase downstream efficiencies. These, in turn, resulted in a continued decrease in LWBS rates. Examples of these additional strategies included implementing team rounding on hospital floors. These teams consisted of physicians, case managers, and unit managers. We also improved door-to-provider time in 2018 and 2019 which led to continued improvement in LWBS.

There were some untoward negative effects of the direct bedding strategy. Health care providers (physicians, physician assistants, Advanced Practice Registered Nurses [APRNs], and nursing staff) all saw their patient loads increased. Some patient care spaces became smaller and less intimate (e.g., hallway spaces and chairs). "Pull-to-full" also created conflicts between nursing and physicians. Nursing protocols mandated swiftly bringing the patients into the department, increasing the number of patients being seen simultaneously by each physician [18]. Not surprisingly, during this time, physician and nursing staff demonstrated lower job satisfaction as reflected by our annual employee engagement surveys. We believe, based on the comments from this instrument that the direct bedding strategy and its increased push of patients into the department, contributed to this decline.

As with other hospitals seeking to improve hospital flow, there were several tactics implemented to help explain the dramatic reduction in LWBS starting in 2015. The ED direct bedding of patients was the most prominent and central strategy for decreasing LWBS. It produced a significant and almost instantaneous decrease in LWBS. It also encouraged other initiatives across the institution to capitalize on our success by also implementing strategies to increase flow through the hospital to discharge. In particular, the ED and hospitalist clinicians became engaged to see patients more quickly than in previous years. Better data collection, frequent rounding in the ED and on the hospital floors, as well as daily collection of discrete data as described above, resulted in a positive, multi-departmental emphasis on reducing LWBS and increasing patient care quality overall.

## Conclusions

We have demonstrated that the implementation of the direct bedding strategy in September 2015, in combination with other initiatives focused on patient flow, demonstrated a profound and sustained association with decreasing LWBS. It is worth noting that although this report focuses on retrospective data during the first two years of our "pull-to-full" initiative, our LWBS rates continue to decline. Currently, we have reduced our overall LWBS rates to under 1%. This, most likely, was a direct result of a comprehensive and multi-disciplinary and inter-departmental cooperative push which began after the positive change implemented by our initial "pull-to-full" policies.
